# Fusarinine C, a novel siderophore‐based bifunctional chelator for radiolabeling with Gallium‐68

**DOI:** 10.1002/jlcr.3286

**Published:** 2015-04-15

**Authors:** Chuangyan Zhai, Dominik Summer, Christine Rangger, Hubertus Haas, Roland Haubner, Clemens Decristoforo

**Affiliations:** ^1^ Department of Nuclear MedicineMedical University InnsbruckInnsbruckAustria; ^2^ Division of Molecular BiologyMedical University InnsbruckInnsbruckAustria; ^3^ Department of Nuclear MedicineGuangdong General Hospital and Guangdong Academy of Medical SciencesGuangzhouChina

**Keywords:** fusarinine C, RGD, ^68^Ga, radiolabeling, positron emission tomography (PET)

## Abstract

Fusarinine C (FSC), a siderophore‐based chelator coupled with the model peptide c(RGDfK) (FSC(succ‐RGD)_3_), revealed excellent targeting properties *in vivo* using positron emission tomography (PET). Here, we report the details of radiolabeling conditions and specific activity as well as selectivity for ^68^Ga.

^68^Ga labeling of FSC(succ‐RGD)_3_ was optimized regarding peptide concentration, pH, temperature, reaction time, and buffer system. Specific activity (SA) of [^68^Ga]FSC(succ‐RGD)_3_ was compared with ^68^Ga‐1,4,7‐triazacyclononane, 1‐glutaric acid‐4,7 acetic acid RGD ([^68^Ga]NODAGA‐RGD). Stability was evaluated in 1000‐fold ethylenediaminetetraacetic acid (EDTA) solution (pH 7) and phosphate‐buffered saline (PBS). Metal competition tests (Fe, Cu, Zn, Al, and Ni) were carried out using [^68^Ga]‐triacetylfusarinine C.

High radiochemical yield was achieved within 5 min at room temperature, in particular allowing labeling with ^68^Ga up to pH 8 with excellent stability in 1000‐fold EDTA solution and PBS. The 10‐fold to 20‐fold lower concentrations of FSC(succ‐RGD)_3_ led to the same radiochemical yield compared with [^68^Ga]NODAGA‐RGD with SA up to 1.8 TBq/µmol. Metal competition tests showed high selective binding of ^68^Ga to FSC.

FSC is a multivalent siderophore‐based bifunctional chelator allowing fast and highly selective labeling with ^68^Ga in a wide pH range and results in stable complexes with high SA. Thus it is exceptionally well suited for the development of new ^68^Ga‐tracers for *in vivo* molecular imaging with PET.

## Introduction

Positron emission tomography (PET) is one of the most prominent molecular imaging modalities, mainly based on the clinical utility of [^18^F]fluorodeoxyglucose. The potential to radiolabel molecules with a high specificity towards a biological target offers tremendous opportunities both for clinical as well as preclinical applications. However, the use of ^18^F and other radionuclides such as ^11^C relies on the availability of cyclotrons in the vicinity of the nuclear medicine department and even as the capacities of production and distribution are increasing; the technology is hampered by the bottleneck of cyclotron‐based radionuclide production. Therefore, the last ten years have seen a tremendous interest in other radionuclides, in particular with improved availability based on generator elution. The most promising candidate is ^68^Ga, having a very suitable half‐life of 68 min, a high positron yield (89%) and therefore can be seen as a metallic alternative to ^18^F.^1^, ^2^

For labeling of molecular probes with ^68^Ga, a coordinating system is required. Therefore, the vector molecule should be conjugated with a bifunctional chelator. In contrast to ^99m^Tc, which is widely used in clinical settings for single photon emission computed tomography and where a great number of structurally different chelating agents are available, less variety can be found for ^68^Ga, even though research in this field is very active increasing the number of chelating systems constantly.^2^ Until now, the most widely applied chelating system for radiolabeling of biomolecules with ^68^Ga is 1,7,7,10‐tetraazacyclododecane‐1,4,7,10‐tetraacetic acid (DOTA). However, DOTA is not ideally suited for Ga^3+^ having an effective ionic radius of 62 pm, comparable to Fe^3+^ (64.5 pm) and Zr^4+^ (72 pm) but considerably smaller than other radiometals used in the context of DOTA labeling such as In^3+^, Y^3+^, or lanthanides (La^3+^) with >80 pm.^3^ An alternative chelating system for ^68^Ga is 1,4,7‐triazacyclononane‐1,4,7‐triacetic acid (NOTA), which has a smaller cavity and allows binding of the Ga^3+^ cation with higher kinetic stability and labeling yield than its corresponding DOTA counterpart. Some triazacyclononane derivatives (e.g., NODAGA,^4^ 1,4,7‐triazacyclononane phosphinic acid (TRAP),^5^ and 1,4,7‐triazacyclononane‐1,4‐bis[methylene(hydroxymethyl)phosphinic acid]‐7‐[methylene(2‐carboxyethyl)phosphinic acid] (NOPO)^6^, ^7^) are increasingly utilized in the development of ^68^Ga tracers.

An alternative to these cyclic aminocarboxylates and aminophosphinates is siderophores. These are low molecular mass chelators with a very high affinity for ferric ions and are utilized by bacteria, fungi, and plants for iron acquisition and storage.^8^ Because of the comparable ionic radius, charge, and electronic configuration, ^68^Ga^3+^ shows almost identical coordination chemistry to Fe^3+^ and can be used to radiolabel the corresponding siderpohore. One of the first siderophores used for labeling with ^67/68^Ga^3+^ was desferoxamine B (DFO) with high radiochemical yield (RCY).^9^ [^67/68^Ga]DFO‐octreotide showed high and specific tumor uptake in mice but disappointing data in patient because of strong protein binding in human plasma.^10^

**Figure 1:**
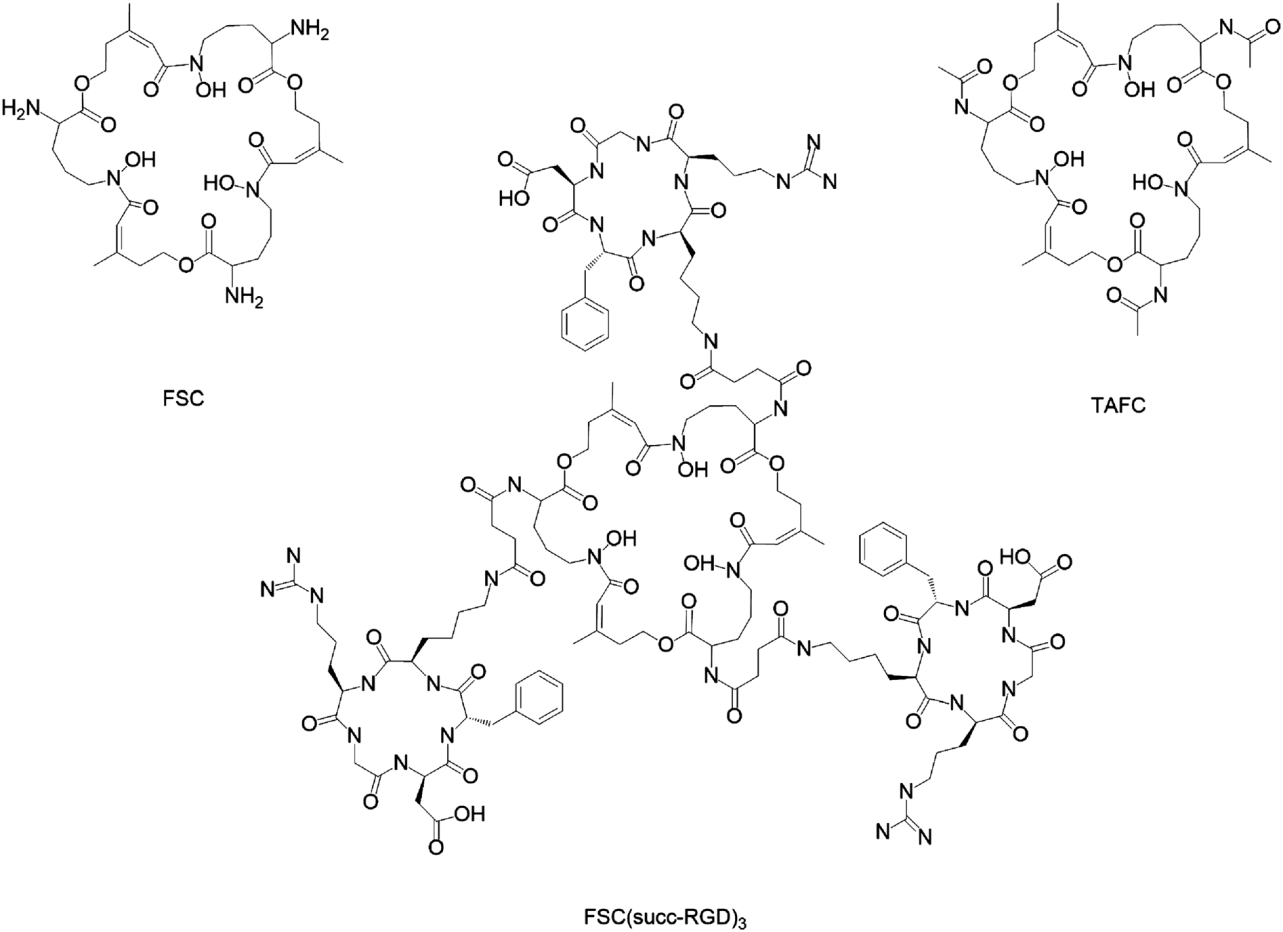
Structures of FSC, TAFC, and FSC(succ‐RGD)_3_.

Recently, we have reported that triacetylfusarinine (TAFC) (Figure 1), which forms hexacoordinate ferric complexes in a tris‐bidentate form involving the three hydroxamate functions of the molecule, had very good complexing properties for ^68^Ga resulting in high specific activities (SAs) and excellent metabolic stabilities.^11^, ^12^ In the light of these promising properties, we were interested whether fusarinine C (FSC), the deacetylated form of TAFC, could be used as a basis for preparing specifically targeted ^68^Ga‐labeled bioconjugates. To evaluate this hypothesis, a RGD peptide targeting the integrin *α*_v_ *β*_3_, which is overexpressed on activated endothelial cells during tumor neovascularization,^13^ was chosen as model targeting molecule. FSC(succ‐RGD)_3_ was synthesized and evaluated *in vitro* and *in vivo*.^14^ Our initial data showed that FSC can be used as bifunctional chelator for synthesis of trimeric bioconjugates allowing ^68^Ga labeling with excellent radiolabeling properties. Moreover, high affinity for integrin *α*_v_ *β*_3_ *in vitro* and high tumor uptake *in vivo* of [^68^Ga]FSC(succ‐RGD)_3_ proved highly promising properties of the FSC chelating scaffold. Here, we report on the optimization of radiolabeling conditions including metal competition tests and achievable SA of [^68^Ga]FSC(succ‐RGD)_3_ and compared them with the established monomeric [^68^Ga]NODAGA‐RGD.^15^

## Materials and methods

All described substances and solvents were of reagent grade and were used without further purification. NODAGA‐RGD was synthesized in our laboratory as described earlier.^15^ [Fe]Fusarinine C ([Fe]FSC) and TAFC were gifts from Prof. Hass and were prepared as described previously.^13^, ^14^ MilliQ water was used for preparing all reagent solutions. The ^68^Ga‐generator (IGG100) was purchased from Eckert & Ziegler Strahlen‐ und Medizintechnik AG (Berlin, Germany) with a nominal activity of 1100 MBq and was eluted with 0.1M HCl solution (Rotem Industries Ltd., Beer‐Sheva, Israel).

### Analytical methods

Analytical reversed‐phase high‐performance liquid chromatography (RP‐HPLC) analysis was performed with an UltiMate 3000 RS HPLC pump, an UltiMate 3000 RS column compartment (column oven temperature was set at 25°C), an UltiMate 3000 ultraviolet (UV)–vis variable wavelength detector (Dionex, Gemering, Germany), and a Raytest radiometric detector (Raytest GmbH, Straubenhardt, Germany). A Vydac 218 TP5215, 150 × 3.0 mm column (SRD, Vienna, Austria), flow rate 1.0 mL/min, and UV–vis detection at 220/410 nm were employed with the following acetonitrile (CH_3_CN)/H_2_O/0.1% trifluoroacetic acid (TFA) gradient: 0–0.5 min 0% CH_3_CN, 0.5–7.0 min, 0–55% CH_3_CN.

Radiolabeling efficiency and radiochemical purity were additionally analyzed by instant thin‐layer chromatography (ITLC) on ITLC silica gel strips with two different mobile phases: 0.1 M aqueous sodium citrate (pH 5) (TLC1) and with a 1:1 (*v*/*v*) mixture of 1 M aqueous ammonium acetate and methanol as a mobile phase (pH 7) (TLC2). The strips were scanned using a mini‐scan radio TLC scanner with a flow‐count detector (LabLogic, Sheffield, UK).

### Synthesis of FSC(succ‐RGD)_3_

Detailed chemical synthesis of FSC(succ‐RGD)_3_ is described in reference.^14^ Briefly, the side‐chain protected c(‐Arg(Pbf)‐Gly‐Asp(OtBu)‐_D_Phe‐Lys(Z)‐) (protecting group: Pbf: 2,2,4,5,7‐pentamethyl‐3‐hydrobenzofuran‐6‐sulfonyl, OtBu: O‐tert‐butyl, Z: benzyloxycarbonyl) was synthesized using a Fmoc protection strategy, followed by cyclization and lysine (Lys) sidechain deprotection. The amino group of Lys was derivatized by succinic anhydride (succ). An excess of c(‐Arg(Pbf)‐Gly‐Asp(OtBu)‐_D_Phe‐Lys(succ)‐) was conjugated with [Fe]FSC without further purification. The side‐chain deprotection using TFA resulted in [Fe]FSC(succ‐RGD)_3_. The removal of iron from [Fe]FSC(succ‐RGD)_3_ was accomplished by dissolving the conjugate in disodium ethylenediaminetetraacetic acid (EDTA) solution (25 mM, pH 4). The crude product was purified via preparative RP‐HPLC and lyophilized. Matrix‐assisted laser desorption ionization time‐of‐flight mass spectrometry [M + H]^+^ = 2785.0 [C_126_H_183_N_33_O_39_; exact mass: 2782.3 (calculated).

### Radiolabeling of FSC(succ‐RGD)_3_ with Ga‐68

For the optimization of the labeling conditions, the following parameters were investigated: peptide concentration, pH, temperature, buffer systems, and time dependencies of radioactivity incorporation. To a fraction of 1 mL of the generator eluate containing the highest activity (approximately 400–500 MBq in 0.1 M HCl), a solution of sodium acetate (NaOAc, 1.9 M) or 4‐(2‐hydroxyethyl) piperazine‐1‐ethanesulfonic acid (HEPES) buffer (1 M) was added and mixed by brief shaking, resulting in a pH ranging from 1.1 to 6. Subsequently, 100 μL of the corresponding solution was mixed with 20 μL of FSC(succ‐RGD)_3_ with concentrations ranging from 0.04 to 45 μM. For pH 7 and pH 8, the radiolabeling protocol was changed slightly: 20 μL of stock solutions of FSC(succ‐RGD)_3_ were mixed with 100 μL ^68^Ga eluate, then HEPES sodium salt solution (1 M) was added to adjust to appropriate pH. The reaction mixtures were incubated at room temperature (RT) or heated to 70°C and subsequently cooled down in a water bath. At different time points (2, 3, 5, 15, and 20 min), 5 μL of the labeling solution was removed to detect the reaction rate. RCYs were determined via analytical RP‐HPLC (*t_R_* = 6.6 min) and Radio‐TLC (TLC1: [^68^Ga]FSC(succ‐RGD)_3_ stayed at the origin, and free ^68^Ga moved with the front; TLC2: ^68^Ga‐colloid stayed at the origin and [^68^Ga]FSC(succ‐RGD)_3_ moved with the front).

### Radiolabeling of NODAGA‐RGD with ^68^Ga

The optimal labeling condition of NODAGA‐RGD with ^68^Ga was adopted according to reference.^15^ Briefly, to a fraction of 1 mL of the generator eluate containing the highest activity (approximately 400–500 MBq in 0.1 M HCl), a solution of NaOAc (1.9 M, 180 μL) was added and mixed by brief shaking, resulting in a final solution with pH 5. Then 100 μL of this solution was mixed with 20 μL NODAGA‐RGD with concentrations ranging from 0.6 to 10 μM. The labeling mixtures were allowed to react for 15 min at RT. RCYs were determined via analytical RP‐HPLC (*t_R_* = 5.3 min).

### Competition experiments

For the investigation of the influence of metals during ^68^Ga‐radiolabeling, the acetylated version of FSC, TAFC was used as model compound and radiolabeled with ^68^Ga adding different metals in concentrations ranging from 3 to 30 000 μM. The metals [Fe(NO_3_)_3_, FeSO_4_, Al_2_(SO_4_)_3_, ZnSO_4_, CuSO_4_, Ni(NO_3_)_3_ and SnCl_2_] were dissolved in 0.1 M HCl to yield stock solutions in a concentration of 84 mM. The stock solutions were diluted to 8.4 mM, 840, 84 and 8.4 μM, respectively. These solutions were mixed with a freshly eluted ^68^Ga fraction (*v*:*v* 1:1). Radiolabeling was performed as described in the preceding texts. The final volume of all labeling solutions was 140 μL with a final TAFC concentration of 3 μM and final metal concentrations ranged from 3 μM to 30 mM, the reactions were carried out at pH 4.5 for 15 min. As a control the influence of Fe^3+^ on ^68^Ga‐labeling of NODAGA‐RGD was tested following the same protocol. RCYs were determined by TLC 1.

### Stability assay

Investigation of the stability of [^68^Ga]FSC(succ‐RGD)_3_ in 1000‐fold EDTA solution (pH 7) was carried out by incubating the radiotracer for 30, 60, and 120 min at 37°C. An aliquot of 100 μL of [^68^Ga]FSC(succ‐RGD)_3_ (50 μM) was mixed with 100 μL of EDTA solution (50 mM). The pH was adjusted to 7. At selected time points 10 μL aliquots were analyzed directly via analytical RP‐HPLC.

Investigation of the stability of [^68^Ga]FSC(succ‐RGD)_3_ at different concentrations were carried out by diluting the radiotracer (50 μM) with PBS to give FSC(succ‐RGD)_3_ concentrations ranging from 50 μM to 5 nM. The samples were analyzed at selected time points (10 and 60 min) via analytical RP‐HPLC.

## Results and discussion

In this study, we focused on the optimization of radiolabeling conditions to reach the highest SA of [^68^Ga]FSC(succ‐RGD)_3_, to determine the stability of [^68^Ga]FSC(succ‐RGD)_3_ under different conditions, and to evaluate the influence of various metals on ^68^Ga labeling.

### Buffer system

**Figure 2:**
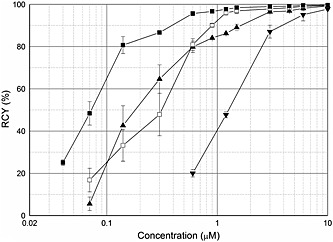
Influence of temperture and buffering system on the radiochemical yield of radioligand. [^68^Ga]FSC(succ‐RGD)_3_: 70°C, NaOAc, pH 4.5 (—■—), RT, NaOAc, pH 4.5 (—□—), RT, HEPES buffer, pH 4.5 (—▲—); [^68^Ga]NODAGA‐RGD: RT, NaOAc, pH 5 (—▼—).

The buffer system can have great influence on ^68^Ga‐labeling yield and thus on SA, which has already been reported.^16^ In our study, sodium acetate solution and HEPES buffer were chosen to compare the influence of the buffer on FSC(succ‐RGD)_3_ labeling. As shown in Figure 2 higher RCYs of [^68^Ga]FSC(succ‐RGD)_3_ were achieved using HEPES buffer at low precursor concentrations (< 0.6 μM) at RT (15 min reaction time). Comparable RCYs for both buffer systems were found at 0.6 μM precursor concentration, but lower RCYs for HEPES at higher precursor concentrations (>0.6 μM). The concentration of precursor required for quantitative ^68^Ga incorporation was 1.2 μM when using sodium acetate and 3 μM in the case of HEPES buffer at RT. At elevated temperature (70°C), quantitative ^68^Ga incorporation was achieved at 0.6 μM precursor concentration using sodium acetate. This indicates an advantage of the sodium acetate systems for ^68^Ga labeling of FSC conjugates in particular at increased temperature.

### Specific activity

**Figure 3:**
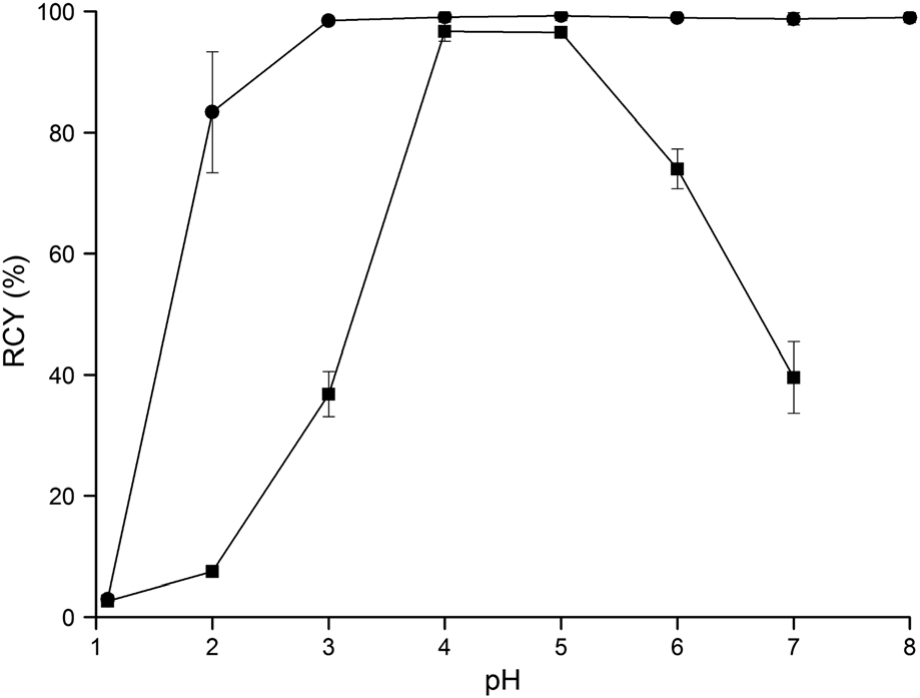
Influence of pH on the radiochemical yield of [^68^Ga]FSC(succ‐RGD)_3_: RT, 45 μM (—●—), RT,1.5 μM (—■—).

We have reported the feasibility of labeling NODAGA‐RGD with ^68^Ga at ambient temperature.^15^ As shown in Figure 3, fairly high concentrations of NODAGA‐RGD are required to achieve quantitative labeling at RT. Comparing RCYs of [^68^Ga]FSC(succ‐RGD)_3_ with [^68^Ga]NODAGA‐RGD approximately 10‐fold to 20‐fold lower precursor concentrations for labeling our FSC conjugate were required, which provided much higher SA because of the lower excess of precursor required for labeling. Table 1 shows the RCY and corresponding calculated SA of [^68^Ga]FSC(succ‐RGD)_3_ and [^68^Ga]NODAGA‐RGD at different precursor concentrations. The SAs and RCYs were calculated by normalizing the starting activities of these set of experiments to approximately 50 MBq in 100‐μL eluate. Considering a reproducible RCY > 80%, a SA up to 1.8 TBq/µmol was observed for [^68^Ga]FSC(succ‐RGD)_3,_ approximately 18‐fold higher than [^68^Ga]NODAGA‐RGD where only 0.1 TBq/µmol were achieved with a RCY of 87%. This allows preparation of ^68^Ga‐labeled tracers targeting low capacity sites for clinical but also preclinical applications, where variation of SA over a wider range may be beneficial to achieve optimal targeting. Additionally regulatory toxicity concerns can be more easily addressed. The maximal achievable SA in this study is close to the extraordinary high SA reported for phosphinic acid functionalized polyazacycloalkanes such as NOPO and TRAP.^17^

**Table 1:** Radiochemical yields and calculated specific activities for [^68^Ga]FSC(succ‐RGD)_3_ and [^68^Ga]NODAGA‐RGD

Precursor amount	Precursor concentration	Radiochemical yield	Specific activity
(nmol)	(μM)	(%)	(GBq/µmol)
[^68^Ga]FSC(succ‐RGD)_3_			
0.0045	0.04	25.1 ± 1.3	2230 ± 118
0.009	0.07	48.4 ± 5.6	2150 ± 247
0.018	0.14	80.7 ± 4.0	1792 ± 89
0.036	0.3	86.6 ± 0.1	962 ± 1.6
0.072	0.6	95.6 ± 1.0	531 ± 5.5
0.11	0.9	96.7 ± 0.2	352 ± 0.7
[^68^Ga]NODAGA‐RGD			
0.072	0.6	20.0 ± 1.8	111 ± 10
0.14	1.2	47.7 ± 1.4	136 ± 4
0.36	3	87.0 ± 3.1	97 ± 3.4
0.72	6	95.1 ± 2.9	53 ± 1.6
1.20	10	97.7 ± 0.4	33 ± 0.1

Values presented as mean ± SD (*n* ≥ 3 of separate labeling experiments) at typical time of injection (30 min after start of labeling), activity used 40 MBq.

### Influence of pH

Gallium adopts oxidation state + III in aqueous solution and acts as a hard Lewis acid with high charge density and small ionic radius, which can form stable complexes with hard Lewis bases, such as hydroxide, alkoxide, halogen, and ammonia moieties. If Ga^3+^ concentration exceeds the nanomolar range in aqueous solution, it can hydrolyze to different insoluble colloidal gallium hydroxide species which occurs at a pH of approximately 5, while at a pH of 8 (or higher) highly soluble [Ga(OH)_4_]^‐^ ions are formed. Radiolabeling procedures with Ga^3+^ are usually carried out at pH 2–4 (e.g., DOTA‐TOC pH 3.5–4.0^18^), whereas labeling at higher pH is also possible (e.g., NODAGA‐RGD at pH 5) depending on the chelating system applied.^19^ Šimeček's group^6^ recently reported that NOPO, a phosphinic acid functionalized polyazacycloalkane, can be quantitatively labeled at pH 7 using 10 μM labeling precursor at 95°C but reported a dramatic decrease of RCY at pH 8. In our study, the influence of pH was investigated at RT (Figure 3), where FSC(succ‐RGD)_3_ in 1.5 μM concentration could be labeled quantitatively at pH 4 and 5. A particularly interesting feature of FSC(succ‐RGD)_3_ is that it can be labeled readily at a substantially higher pH values using a higher concentration of precursor. In our study, quantitative labeling was achieved using 45 μM precursor concentration at pH 7 and 8, respectively. The advantage of radiolabeling at pH 7 and 8 is the potential to radiolabel precursors with ^68^Ga that only dissolve in neutral or basic solutions or are unstable at acidic pH levels.

### Influence of reaction time

**Figure 4:**
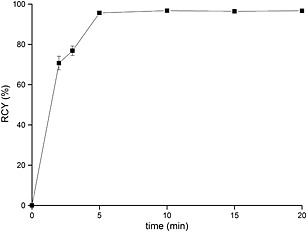
Influence of reaction time on the radiochemical yield of [^68^Ga]FSC(succ‐RGD)_3_ (1.5 μM, RT, pH 4.5).

^68^Ga, with a short half‐life of 68 min, also requires minimizing radiolabeling time. Figure 4 shows the influence of the reaction time on the RCY of [^68^Ga]FSC(succ‐RGD)_3_. A RCY of 95.7% was achieved within 5 min at pH 4.5 at RT and did not increase with prolonged reaction time.

### Metal ions

**Figure 5:**
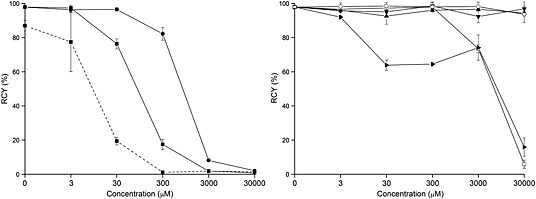
Influence of metals on TAFC and NODAGA‐RGD labeling with ^68^Ga. Left: [^68^Ga]FSC(succ‐RGD)_3_: Fe(NO_3_)_3_ (—■—), FeSO_4_ (—●—), [^68^Ga]NODAGA‐RGD: Fe(NO_3_)_3_ (‐‐‐■‐‐‐); Right: [^68^Ga]FSC(succ‐RGD)_3_: Ni(NO_3_)_3_ (—▼—), SnCl_2_ (—▸—), Al_2_(SO_4_)_3_ (—□—), ZnSO_4_ (—▲—), CuSO_4_ (—○—).

The influence of metal ions on ^68^Ga labeling of chelators like NOTA, DOTA, TRAP‐Pr, or NOPO has been studied by Šimeček et al.^20^ TAFC was used for metal competition experiments in our study as a model FSC‐derivative. The influence of different concentrations of metal ions (Fe^3+^, Fe^2+^, Al^3+^, Zn^2+^, Cu^2+^, Ni^3+^, and Sn^2+^) on ^68^Ga labeling of TAFC was studied, as comparison for the influence of Fe^3+^ NODAGA‐RGD was used (Figure 5). Labeling was performed at 3‐μM precursor concentration to ensure quantitative reaction conditions. Particular focus was put on iron as a very common metal impurity in generator eluates but also as FSC and TAFC are siderophores with high affinity for iron. We found no influence on labeling of TAFC even if the concentration of Fe^3+^ was equal to the concentration of TAFC and the concentration of ^68^Ga^3+^ much lower in the labeling solution. This indicates a higher affinity of FSC for Ga^3+^ possibly attributed to the harder Lewis acid property of Ga^3+^. At higher concentrations, RCY dropped significantly; for Fe^2+^, this effect on ^68^Ga labeling was less pronounced; quantitative labeling could still be achieved with 30 μM of Fe^2+^ concentration, 10 times higher than that of Fe^3+^. In contrast to TAFC, NODAGA‐RGD labeling with ^68^Ga was influenced by the presence of Fe^3+^, which was reflected by a greater variation in RCYs at the concentration of 3 μM Fe^3+^ and a dramatic RCY decrease at higher concentration. Al^3+^, with a trivalent charge and a proximity to Ga in the periodic table, had no influence on ^68^Ga labeling of TAFC up to a concentration of 300 μM. Zn^2+^ as the decay product of ^68^Ga accumulating in the generator did not decrease RCYs even at the concentration of 30 mM, which is highly unlikely to occur in a generator. Cu^2+^ and Ni^3+^ were also studied but had no influence on ^68^Ga labeling. Sn^2+^ was found to have a minor effect on ^68^Ga labeling at 3 μM, RCYs decrease to approximately 64% at 30 μM and remained stable up to 3 mM. Overall FSC‐derivatives have an excellent selectivity for ^68^Ga superior to NOTA‐derivatives and comparable to triazacyclononane–phophinates as reported previously.^6^, ^7^

### Stability

We already reported that [^68^Ga]FSC(succ‐RGD)_3_ was stable in PBS, FeCl_3_, DTPA solution and in fresh human serum at 37°C for 2 h.^16^ We additionally evaluated the stability of [^68^Ga]FSC(succ‐RGD)_3_ in EDTA solution because iron was removed quickly from [Fe]FSC(succ‐RGD)_3_ in 40‐fold EDTA solution at pH 4 during the synthesis of the conjugate. In our study, 1000‐fold EDTA was mixed with [^68^Ga]FeFSC(succ‐RGD)_3_ at pH 7 and analyzed at 30, 60, and 120 min with a HPLC system. There was almost no detectable decrease of RCY during the whole monitoring period, indicating a high stability at neutral pH.

Caraco et al. reported that dilution of DFO, an acyclic analogue of FSC, could cause the breakdown of the ^67^Ga^3+^–DFO complex. High RCYs can be achieved at concentrations >5 mM, while at nanomolar level (<50 nM) DFO does not act as a good chelating agent for gallium.^21^ In our study, 50 μM of [^68^Ga]FSC(succ‐RGD)_3_ was diluted in PBS to obtain FSC(succ‐RGD)_3_ in concentrations ranging from 50 μM to 5 nM and was analyzed at different time points (10 and 60 min after dilution) by RP‐HPLC. No dissociation of ^68^Ga from the FSC chelator was observed over 60 min, even at the lowest [^68^Ga]FSC(succ‐RGD)_3_ concentration, indicating superior stability to that of DFO particularly at low precursor concentrations.

## Conclusion

The radiolabeling conditions and stabilities of the new siderophore‐based bifunctional chelator FSC coupled to the model peptide RGD were studied and showed that high RCY was achieved within 5 min at RT and within a wide pH range (3–8). In particular labeling at pH 7 and 8 was possible providing access to ^68^Ga‐labeled biomolecules that are unstable at acidic conditions. Specific activity achieved was 10–20 times higher compared with [^68^Ga]NODAGA‐RGD and was comparable to TRAP or NOPO conjugates. Excellent stability towards 1000‐fold EDTA challenge and PBS dilution at pH 7 was observed and metal competition tests showed high selectivity of FSC for complexing ^68^Ga. We conclude that FSC is exceptionally well suited for the development of ^68^Ga‐labeled tracer for *in vivo* molecular imaging with PET providing a high flexibility in labeling conditions and is an interesting alternative to currently used triazacyclononanes. The structural relationship to DFO also raises the possibility of using FSC for radiolabeling with ^89^Zr [28]. Investigations in this direction are ongoing.

## Conflict of Interest

The authors have declared that there is no conflict of interest.
